# P-1683. Breathless Battles: molecular microbiology profile of low respiratory tract infections in a tertiary care hospital

**DOI:** 10.1093/ofid/ofaf695.1857

**Published:** 2026-01-11

**Authors:** Pamela Machado, Ann S Sánchez-Marmolejos, Lia Chaddy-Baéz, Anel E Guzmán-Marte, Florangel Grullón, José A Ledesma-Baéz, Osvaldo D Cabrera-Castellanos

**Affiliations:** Hospital General De La Plaza De La Salud, Distrito Nacional, Distrito Nacional, Dominican Republic; Hospital General de la Plaza de la Salud, Distrito Nacional, Distrito Nacional, Dominican Republic; Hospital General De La Plaza De La Salud, Distrito Nacional, Distrito Nacional, Dominican Republic; Hospital General De La Plaza De La Salud, Distrito Nacional, Distrito Nacional, Dominican Republic; Hospital General De La Plaza De La Salud, Distrito Nacional, Distrito Nacional, Dominican Republic; Hospital General De La Plaza De La Salud, Distrito Nacional, Distrito Nacional, Dominican Republic; Hospital General Plaza de la Salud, santo domingo, Distrito Nacional, Dominican Republic

## Abstract

**Background:**

Pneumonia-related infections remain a frequent challenge in hospitalized patients due to their complex causes, severity, and increasing antimicrobial resistance. This study aimed to describe the clinical characteristics, microbiological findings, and resistance gene patterns in patients tested with a pneumonia multiplex panel at a tertiary hospital in the Dominican Republic.

**Figure:**
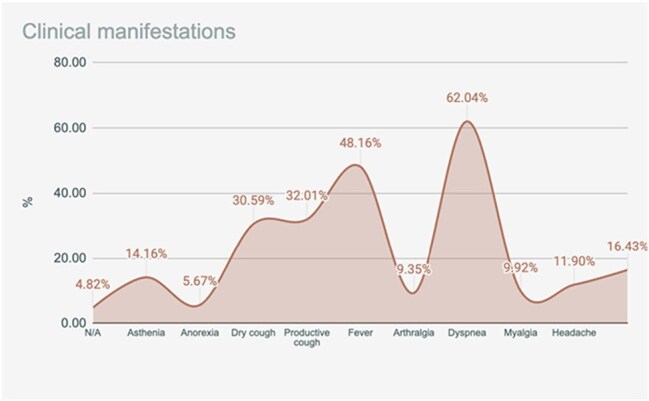

**Figure:**
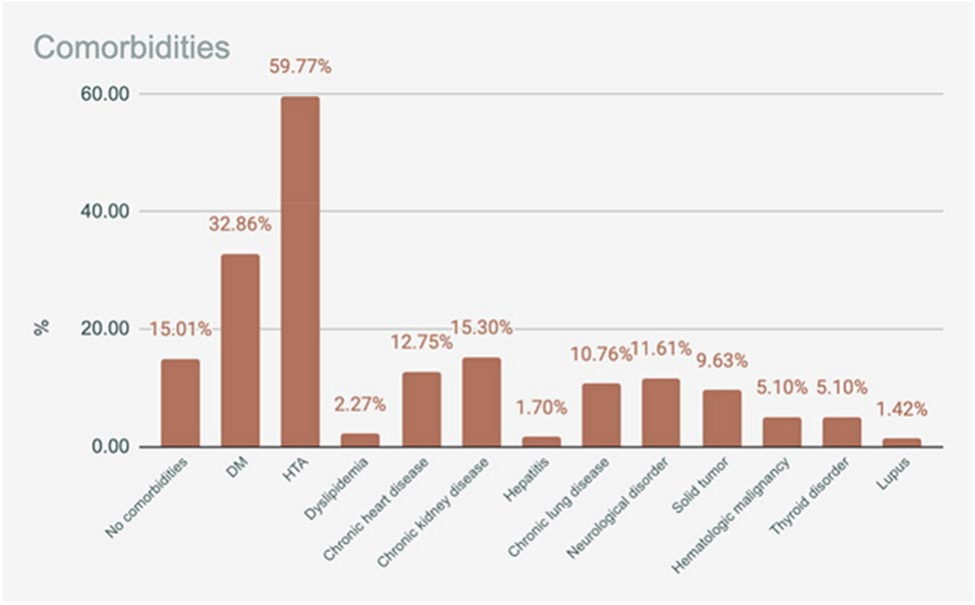

**Methods:**

We conducted a retrospective analysis of 353 patients tested with a Filmarray pneumonia panel between 2020 and 2024. The data collected included demographics, comorbidities, symptoms, ICU admission, microbiological results, and detection of resistance genes.

**Figure:**
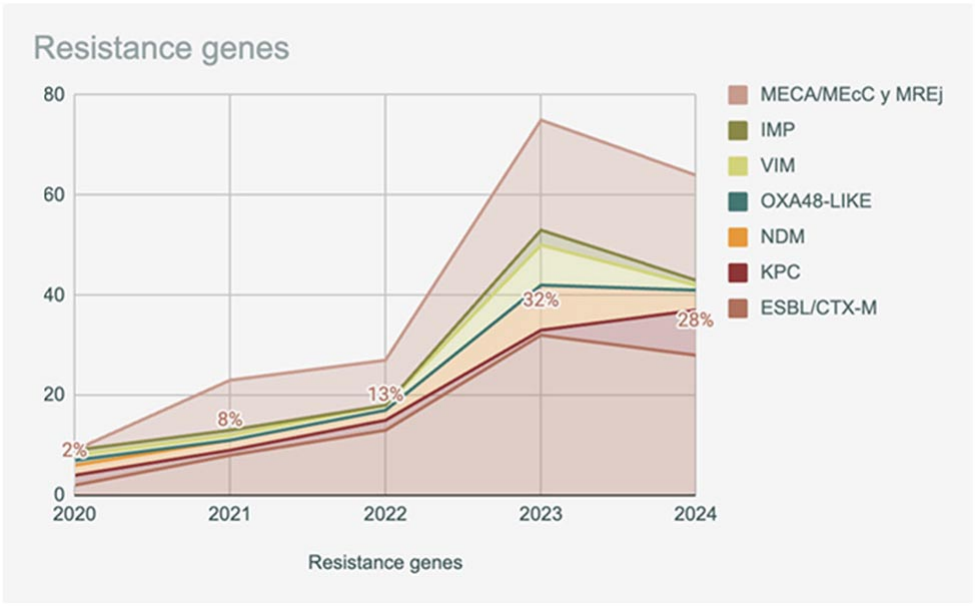

**Results:**

Among 353 patients, 98% were adults and 56.60% were male. The most frequent symptoms were dyspnea (62%), fever (48%), and productive cough (32%). Common comorbidities included hypertension (59.8%), diabetes mellitus (32.9%), and chronic heart disease (12.8%). A total of 187 patients (23%) required ICU care. 32% died, and 11% were readmitted within 30 days.

Bacteria were the most common pathogens, led by *Staphylococcus aureus* (22.4%), *Klebsiella pneumoniae* complex (19.3%), and *Pseudomonas aeruginosa* (17%). Resistance genes were frequently identified: ESBL/CTX-M (23.5%), mecA/mecC + MREJ (17.6%), and NDM (5.4%). Viral agents were also detected, including coronaviruses (11.4%), Rhinovirus/Enterovirus (10.9%), and Influenza A (7%).

**Conclusion:**

The high frequency of severe pneumonia cases, many affecting patients already living with chronic conditions, reveals the considerable clinical burden of this infection in hospitalized populations. The frequent need for intensive care and the high mortality rate reflects the critical importance of early identification and appropriate management. The detection of multidrug-resistant bacteria—often carrying more than one resistance gene—adds complexity to an already fragile clinical picture. These findings highlight the need to strengthen diagnostic capacity and antibiotic stewardship, not only to improve individual outcomes but to safeguard the effectiveness of therapies for future patients.

**Disclosures:**

All Authors: No reported disclosures

